# TLR4 rs2149356 Polymorphism in Periodontitis and End-Stage Renal Disease: An Exploratory Analysis in Egyptian Patients

**DOI:** 10.3390/cells14181421

**Published:** 2025-09-11

**Authors:** Asmaa Abou-Bakr, Fatma E. A. Hassanein, Nermeen Nagi, Mihad Ibrahim, Mohamed Mekhemar

**Affiliations:** 1 Oral Medicine and Periodontology, Faculty of Dentistry, Galala University, Suez 43511, Egypt; asmaa.abdalraouf@gu.edu.eg; 2 Oral Medicine, Periodontology, and Oral Diagnosis, Faculty of Dentistry, King Salman International University, El Tur 46511, Egypt; 3 Department of Fixed Prosthodontics, Faculty of Dentistry, Fayoum University, Fayoum 63514, Egypt; dr.nermeennagi@hotmail.com; 4 Department of Fixed Prosthodontics, Faculty of Dentistry, Galala University, Suez 43511, Egypt; 5 Oral Medicine and Periodontology, Faculty of Dentistry, Cairo University, Giza 12613, Egypt; mihad.ibrahim@dentistry.cu.edu.eg; 6 Clinic for Conservative Dentistry and Periodontology, School of Dental Medicine, Christian-Albrecht’s University, 24105 Kiel, Germany

**Keywords:** Toll-like receptor 4, periodontitis, ESRD, inflammation, genetic polymorphism, microbiota, gene–environment interaction

## Abstract

This study explored the association of the TLR4 rs2149356 polymorphism with periodontal and renal parameters in Egyptian end-stage renal disease (ESRD) patients. Ninety-two patients with periodontitis were recruited, forty-six on hemodialysis, and forty-six systemically healthy controls. Clinical periodontal indices, renal biomarkers, and gGenotyping for TLR4 rs2149356 were assessed. Gingival inflammation was significantly higher in ESRD patients across all genotypes. Although the TT genotype showed a trend toward deeper probing depths and greater attachment loss in ESRD patients, these differences did not reach statistical significance after correction. Regression models indicated that TT carriers exhibited higher inflammatory and renal burden, suggesting a potential gene–environment interaction. TLR4 rs2149356 polymorphism may modulate inflammatory response in ESRD and periodontitis patients, although findings remain exploratory. These results highlight the potential role of host–microbe–gene interactions in systemic inflammation, warranting longitudinal and functional studies in larger, multi-ethnic cohorts.

## 1. Introduction

End-stage renal disease (ESRD) represents the terminal stage of chronic kidney disease (CKD) and is characterized by irreversible loss of renal function, requiring renal replacement therapy through dialysis or kidney transplantation. Globally, CKD affects more than 10% of the adult population, and the prevalence of ESRD continues to increase due to the rising burden of diabetes, hypertension, and aging populations [1,2,3,4]. In addition to its systemic complications, ESRD is associated with profound immune dysregulation, oxidative stress, and a persistent pro-inflammatory state, factors that can also influence oral health [5,6].

Periodontitis is a chronic inflammatory disease of the tooth-supporting structures, initiated by dysbiotic microbial biofilms and sustained by an exaggerated host immune response [7]. The disease leads to progressive destruction of the periodontal ligament and alveolar bone, ultimately resulting in tooth loss if untreated [7]. Its prevalence and severity are markedly higher among ESRD patients than in healthy individuals [8]. The relationship between ESRD and periodontitis is bidirectional, as systemic inflammation, altered immune function, and metabolic disturbances in ESRD can exacerbate periodontal destruction, while periodontitis may contribute to systemic inflammatory load and worsen renal outcomes [6,9]. Elevated levels of pro-inflammatory cytokines such as interleukin (IL)-1β, IL-6, tumor necrosis factor-alpha (TNF-α), and C-reactive protein (CRP) are found in both diseases, suggesting overlapping pathophysiological mechanisms [9].

Toll-like receptors (TLRs) are key components of the innate immune system, recognizing pathogen-associated molecular patterns (PAMPs) and damage-associated molecular patterns (DAMPs) to initiate inflammatory signaling pathways [10]. Toll-like receptor 4 (TLR4) is particularly relevant in this context, as it recognizes lipopolysaccharides (LPS) from Gram-negative bacteria and endogenous ligands released during tissue injury [11]. Upon activation, TLR4 triggers NF-κB-mediated transcription of pro-inflammatory cytokines, contributing to host defense but also to chronic inflammation when dysregulated [11]. In the oral cavity, TLR4 plays a central role in the inflammatory response to subgingival microbiota [12], while in the kidney, it has been implicated in promoting inflammation and fibrosis [13].

Genetic polymorphisms in TLR4 can influence receptor expression, ligand affinity, and downstream signaling, thereby modulating susceptibility to inflammatory diseases [14]. Due to their relative rarity in non-European populations, the extensively researched non-synonymous variants Asp299Gly (rs4986790) and Thr399Ile (rs4986791) have limited global relevance [15,16]. While the intronic single-nucleotide polymorphism (SNP) rs2149356 has gained recent attention since it may control TLR4 gene expression by altering transcription factor binding sites or mRNA splicing [15]. Crucially, a prior study conducted in Egypt found that while rs2149356 was present and substantially linked to periodontitis, rs4986790 and rs4986791 were not found in this population [17]. The most relevant TLR4 variant for exploring inflammatory comorbidity in Egyptians is therefore rs2149356.

Beyond periodontitis, previous studies have associated rs2149356 with inflammatory disorders such as gout [15,18], atherosclerosis [19], and metabolic conditions [20], suggesting a more extensive function in immune-mediated disorders. This increases the likelihood that this viable variant is worth investigating for its potential role in oral–renal inflammatory comorbidity, a relationship that has not yet been explored.

Given the overlapping inflammatory pathways in ESRD and periodontitis [9] and the central role of TLR4 in microbial sensing, the rs2149356 SNP offers a biologically plausible link between genetic predisposition and host–microbe interactions. To date, no studies have examined the association of TLR4 rs2149356 with periodontal status in ESRD patients, particularly in Middle Eastern populations, who are under-represented in genetic epidemiology.

This cross-sectional study aimed to investigate whether the TLR4 rs2149356 polymorphism is associated with periodontal disease severity and renal parameters in Egyptian patients with ESRD. By integrating clinical, biochemical, and genetic data, we explore the potential gene–environment–microbiota interactions underlying systemic inflammation in this high-risk population.

## 2. Materials and Methods

### 2.1. Study Design and Setting

This analytical cross-sectional study was conducted to explore the potential association between TLR4 rs2149356 polymorphism and the severity of periodontitis among Egyptian patients with and without end-stage renal disease (ESRD). Participants were recruited from two sites: the Hemodialysis Unit at Benha University Hospital and the Periodontology Clinic at the Faculty of Dentistry, Fayoum University. The study protocol was registered at ClinicalTrials.gov (NCT06755372) and approved by the Research Ethics Committee at the Faculty of Dentistry, Fayoum University (Approval No. R-625).

### 2.2. Sample Size and Power Calculation

A priori power analysis was conducted to estimate the required sample size based on prior data reporting TT genotype frequencies of approximately 30% in patients with systemic inflammatory diseases [15] and 8% among Egyptian controls with periodontitis [17]. Using a two-proportion test with a two-sided α = 0.05 and equal group sizes, n = 46 per group (92 total), offers about 80% power to detect a difference in TT frequency from 8% (controls) to ≈30% (cases). For continuous periodontal outcomes (such as clinical attachment level and probing depth), the study has 80% power to detect a moderate-to-large effect with a standardized mean difference (Cohen’s d) of ≈0.59 under the same sample size. Standard formulas for two independent proportions and two-sample t-tests were used to calculate power. As a result, the sample size is in line with effect sizes documented in previous rs2149356 research and strikes a balance between practical recruitment limitations and statistical power.

### 2.3. Participant Recruitment and Grouping

The study included a total of 92 unrelated Egyptian adults diagnosed with periodontitis who were divided into two separate, equal-sized cohorts:

Group I (n = 46): ESRD patients on maintenance hemodialysis, with stage II or III periodontitis [21].

Group II (n = 46): Systemically healthy controls with stage II or III periodontitis [21].

Eligibility criteria:

Inclusion criteria:

Group I (ESRD with periodontitis):

1- Egyptian adults ≥ 18 years with a glomerular filtration rate of less than 15 mL/min, indicating ESRD, and were on renal hemodialysis for six to twenty-four months [22].
2- Unrelated Egyptians residing in the same geographical area and suffering from Periodontitis stage II and/or stage III [17].
3- Only patients with controlled hypertension.

Group II (Healthy subjects with periodontitis):

1- Systemically healthy adults ≥ 18 years who have stage II and/or stage III periodontitis
2- No history of systemic diseases or long-term medication use.

Exclusion criteria for both groups:

1- Patients with diabetes mellitus.
2- Smoking habits (current or former).
3- Other systemic disorders, like autoimmune or systemic inflammatory diseases, chronic liver disease, malignancies, and chronic infections (e.g., hepatitis B/C, HIV).
4- Pregnancy or lactation.
5- Patients who had received periodontal therapy in the past six months.

**Figure 1:**
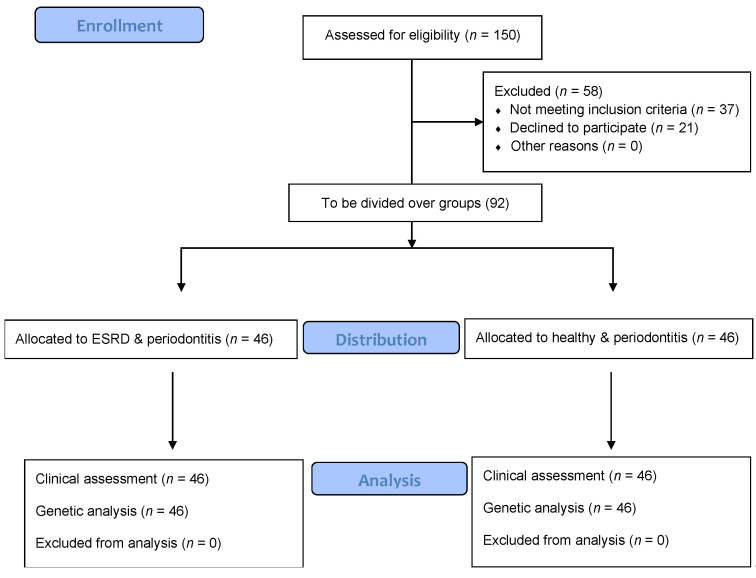
Flow chart for participants’ recruitment.

The flow chart for participant recruitment is illustrated in Figure 1.

### 2.4. Sociodemographic Data

Sociodemographic data (age, sex, education, and income) were collected through structured interviews using a standardized questionnaire. These variables were considered as potential confounders in the association between genetic variation and clinical outcomes.

### 2.5. Clinical Periodontal Assessment

Clinical periodontal examinations were performed by two calibrated examiners [A.A. and F.H.] who were blinded to the participants’ genotypic information and group assignment (ESRD or control). The calibration procedure included: A training session in which both examiners went over the 2018 Classification of Periodontal and Peri-Implant Diseases and Conditions [21] diagnostic criteria as well as standardized probing methods (such as probe insertion angle and force). Trained examiners independently assessed the periodontal clinical parameters (GI, PI, BOP, PD, CAL). Inter-examiner reliability was assessed prior to the study using intra-class correlation coefficients (ICC) for continuous variables and Cohen’s kappa (κ) for categorical measures to ensure consistency.

The following parameters were recorded at six sites per tooth (mesiobuccal, mid-buccal, distobuccal, mesiolingual, mid-lingual, and distolingual) using a Williams periodontal probe: probing depth (PD), clinical attachment level (CAL), plaque index (PI) [23], gingival index (GI) [24], bleeding on probing (BOP%), and plaque coverage (%). PD was defined as the distance from the free gingival margin to the base of the sulcus/pocket. CAL was defined as the distance from the cementoenamel junction (CEJ) to the base of the pocket, with loss of attachment considered present when the probe passed apical to the CEJ. PI was calculated as the proportion of tooth surfaces with visible plaque deposits at the dentogingival junction relative to all examined surfaces, while plaque coverage (%) reflected the percentage of plaque-positive surfaces. GI was scored according to Löe and Silness (1963) [24], grading gingival inflammation by color, edema, and bleeding upon gentle probing. BOP% was assessed dichotomously 10 s after probing with controlled force and expressed as the percentage of bleeding sites per individual. Diagnosis and staging were based on the 2018 Classification of Periodontal and Peri-Implant Diseases and Conditions [21].

### 2.6. Renal Parameters

For Group I, serum creatinine and blood urea nitrogen were measured as standard biochemical markers of renal function [25]. All blood samples were drawn before dialysis sessions to minimize variability.

### 2.7. Genotyping of TLR4 rs2149356

Peripheral blood (5 mL) was collected in EDTA tubes. Genomic DNA was extracted using the QIAamp DNA Mini Kit (QIAGEN, Hilden, Germany), following the manufacturer’s protocol. Genotyping of the rs2149356 SNP was performed as previously described [26] using TaqMan™ allelic discrimination assays (Applied Biosystems, Cat. No. 4351379) in a 25 μL reaction containing TaqMan™ Universal PCR Master Mix. The amplification protocol included an initial denaturation at 96 °C for 10 min, followed by 45 cycles at 60 °C for 90 s and 92 °C for 15 s. Fluorescence was read in a QuantStudio 5 real-time PCR system. Technicians were blinded to the participant’s clinical status.

### 2.8. Statistical Analysis

Analyses were conducted using Stata v18.0 (StataCorp, College Station, TX, USA). Data distribution was assessed using the Shapiro–Wilk test and visual inspection of histograms. Continuous variables were compared with Student’s t-test or Mann–Whitney U test, as appropriate, while categorical variables were assessed with chi-square or Fisher’s exact tests. Inter-examiner reliability was evaluated using intra-class correlation coefficients (ICC, two-way random effects, absolute agreement) for continuous periodontal parameters (probing depth, plaque percentage, bleeding on probing percentage, and clinical attachment level) and weighted Cohen’s kappa (κ) for the ordinal gingival index. Genotype distributions were tested for Hardy–Weinberg equilibrium (HWE) using a chi-square goodness-of-fit test (1 df), and Wright’s fixation index (FIS) was calculated as an effect size measure.

Genotype group comparisons were performed using one-way ANOVA or Kruskal–Wallis tests, depending on distributional assumptions. Multivariable regression models (linear and logistic) were adjusted for age, sex, education, and oral hygiene frequency to control for potential confounders. To account for multiple testing, *p*-values were corrected using the Benjamini–Hochberg false discovery rate (FDR) procedure, with a two-tailed FDR-adjusted *p* ≤ 0.05 considered statistically significant. Effect estimates are presented as regression coefficients (β) or odds ratios (OR) with 95% confidence intervals.

## 3. Results

### 3.1. Sociodemographic Characteristics

The ESRD and control groups were comparable in age, gender distribution, educational level, and financial income, with no statistically significant differences (all *p* > 0.1), indicating successful group matching and minimal risk of sociodemographic confounding in subsequent analyses (Table 1).

### 3.2. Clinical, Biochemical, and Genotypic Characteristics

ESRD patients showed significantly higher gingival inflammation compared to controls (GI: 2.76 ± 0.49 vs. 1.96 ± 0.49; *p* < 0.001). No significant differences were observed in plaque percentage, BOP, or CAL. Dialysis duration averaged 11.1 ± 3.5 months. Serum creatinine and blood urea levels confirmed advanced renal dysfunction in the ESRD group. Regarding periodontitis staging, 12 of 45 ESRD patients (26.7%) and 17 of 46 controls (37.0%) were classified as Stage II, while 33 of 45 ESRD patients (73.3%) and 29 of 46 controls (63.0%) were classified as Stage III; this distribution did not differ significantly between groups (χ^2^ = 0.69, *p* = 0.41). When stratified by genotype, no significant differences were observed in staging distributions between ESRD and controls for GG or GT carriers, whereas TT carriers in the ESRD group demonstrated a significantly higher frequency of Stage III periodontitis compared with controls (*p* < 0.001).

Genotype distribution of TLR4 rs2149356 differed between groups, with the GG genotype more frequent in ESRD patients (12/45, 26.67%) compared to controls (4/46, 8.7%). However, this difference did not remain significant after FDR correction (adjusted *p* = 0.118) (Table 2). The distribution of TLR4 rs2149356 genotypes among the study population was as follows: GG = 4, GT = 16, and TT = 26. The corresponding allele frequencies were 26% for G and 74% for T. Expected counts under Hardy–Weinberg equilibrium (HWE) were GG = 3.13, GT = 17.74, and TT = 25.13. A chi-square test indicated no significant deviation from HWE (χ^2^ = 0.44, *p* = 0.506). The fixation index (FIS) was 0.098, suggesting a minor, non-significant deficit of heterozygotes. These findings confirm that the genotype distribution is consistent with equilibrium expectations, supporting the validity of the genotyping data.

### 3.3. Allelic Comparison

The G allele appeared more frequently in ESRD patients (37/90, 41%) compared to controls (24/92, 26%), but this difference also lost statistical significance after FDR correction (adjusted *p* = 0.093). These results indicate a possible trend, but not conclusive evidence, of allelic association (Table 3).

### 3.4. Relative Risk Analysis

The TT genotype (20/45, 44.4% in ESRD vs. 26/46, 56.5% in controls) conferred a significantly higher relative risk for ESRD compared to the GG genotype (12/45, 26.7% in ESRD vs. 4/46, 8.7% in controls; RR = 3.25, adjusted *p* = 0.020). The GT genotype (13/45, 28.9% in ESRD vs. 16/46, 34.8% in controls) showed a borderline increase in risk (adjusted *p* = 0.060) (Table 4). These results suggest a possible genetic predisposition for ESRD in TT carriers.

### 3.5. Periodontal Parameters by Genotype

Gingival Index (GI) was significantly higher in ESRD patients compared to controls across all genotypes GG (12/45, 26.7% ESRD vs. 4/46, 8.7% controls), GT (13/45, 28.9% vs. 16/46, 34.8%), and TT (20/45, 44.4% vs. 26/46, 56.5%) with FDR-adjusted *p* < 0.05, indicating a consistent effect of renal status on inflammation. No genotype-specific differences in GI were observed within either group.

**Figure 2:**
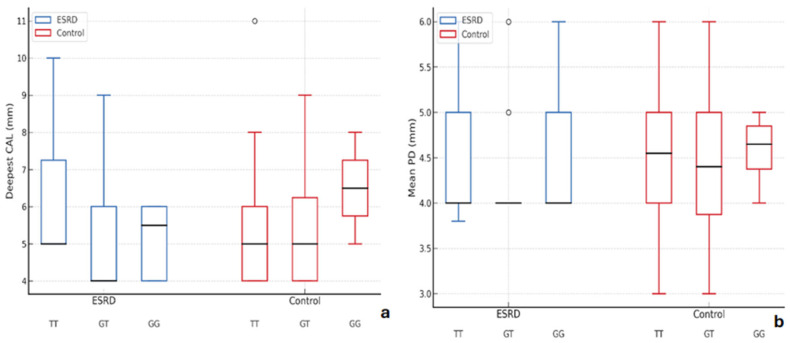
Periodontal parameters distribution according to TLR4 rs2149356 genotypes in ESRD and control groups. (**a**) Box and whisker plot illustrating the distribution of deepest clinical attachment loss (CAL, mm) across TLR4 genotypes (TT, GT, GG) within ESRD and control groups. (**b**) Box and whisker plot showing the mean probing depth (PD, mm) for each TLR4 genotype (TT, GT, GG) in ESRD and control groups. Boxplot elements as in (**a**).

Probing depth (PD) and clinical attachment level (CAL) were higher among ESRD patients with the TT genotype (20/45, 44.4%) compared to controls (26/46, 56.5%) (FDR-adjusted *p* ≈ 0.066), suggesting a potential but not statistically robust association. Other periodontal parameters (PI, plaque %, BOP) did not show significant differences across genotypes (Table 5, Figure 2).

### 3.6. Multivariable Regression Models

In regression models adjusted for age and sex, TT carriers (20/45 ESRD patients, 44.4%; 26/46 controls, 56.5%) were associated with significantly greater PD (β = 1.40; *p* = 0.033), CAL (β = 1.13; *p* = 0.033), and higher GI scores (β = 0.90; *p* < 0.001) compared to GG carriers (12/45 ESRD patients, 26.7%; 4/46 controls, 8.7%). The TT group also showed elevated renal markers: creatinine (β = 0.31; *p* = 0.012) and blood urea (β = 0.27; *p* = 0.009), supporting a potential genetic contribution to combined periodontal and renal burden in TT carriers (Table 6).

### 3.7. Inter-Examiner Reliability

Excellent inter-examiner reliability was demonstrated for Mean PD, Plaque %, BOP %, and deepest Excellent inter-examiner reliability was demonstrated for mean probing depth (PD), plaque percentage, bleeding on probing (BOP) percentage, and deepest clinical attachment level (CAL), with ICC values consistently above 0.95 (*p* < 0.001), indicating strong agreement between examiners for continuous periodontal parameters. For the gingival index (mean), reliability was also high, with a weighted Cohen’s kappa of 0.886 (95% CI: 0.842–0.918, *p* < 0.001), reflecting substantial to almost perfect agreement for this ordinal index. Collectively, these findings confirm robust examiner calibration across both objective and subjective periodontal measures (Table 7).

## 4. Discussion

The present exploratory case–control analysis sought to elucidate potential associations between the TLR4 rs2149356 polymorphism, end-stage renal disease (ESRD), and periodontal status. Our principal findings were threefold: (i) genotype distributions did not differ significantly between ESRD and control groups after false-discovery rate (FDR) correction; (ii) the TT genotype was associated with a higher relative risk for ESRD compared to GG; and (iii) adjusted regression models indicated that TT carriers exhibited more pronounced gingival inflammation, greater probing depth, and increased clinical attachment loss, along with elevated renal biochemical markers. Although these results are not confirmatory, they suggest a biologically coherent pattern that aligns with current mechanistic understanding of TLR4 in inflammatory diseases.

In addition to periodontitis, rs2149356 has been linked in the past to metabolic disorders [20], atherosclerosis [19], and inflammatory diseases like gout [15,18]. This pattern aligns with TLR4′s known role in innate immunity-driven systemic diseases like diabetes mellitus and psoriasis. Self-ligand-induced TLR4 activation in psoriasis promotes keratinocyte proliferation and the chronic inflammatory loop [27]. The chronic low-grade inflammation and insulin resistance that characterize diabetes are also largely mediated by TLR4 signaling [28,29,30].

It is mechanistically plausible that the TLR4 rs2149356 TT genotype and elevated inflammatory burden in ESRD patients are related. In addition to periodontal pathogens [12], endogenous DAMPs and uremic toxins that build up in renal failure [5,6,13] also trigger TLR4 activation. Despite being intronic, the rs2149356 polymorphism may increase TLR4 responsiveness [15,16], resulting in a vicious cycle wherein the uremic milieu and a dysbiotic periodontal biofilm work together to sustain inflammation through persistent NF-κB activation. A framework for gene-environment-microbiota interaction in oral-renal crosstalk is thus provided by the increased systemic cytokines that worsen renal endothelial dysfunction and fibrosis, as well as periodontal destruction [5,9,13]. Comparable findings have been reported in oncology populations, where head and neck cancer patients receiving radiotherapy demonstrated a periodontitis prevalence of 95.08%, with Stage IV being the most common [31].

Toll-like receptor 4 is an archetypal pattern recognition receptor recognizing Gram-negative bacterial lipopolysaccharide and a range of endogenous danger-associated molecular patterns [10,11,12]. Upon activation, TLR4 initiates a MyD88- and TRIF-dependent signaling cascade culminating in NF-κB activation and transcription of pro-inflammatory cytokines, chemokines, and matrix metalloproteinases [11,12]. In the renal compartment, TLR4 expression on tubular epithelial and glomerular endothelial cells is upregulated in CKD and ESRD, where it promotes inflammatory infiltration, oxidative stress, and fibrogenesis [13]. In the periodontium, TLR4 is expressed by epithelial cells, fibroblasts, and periodontal ligament stem cells, and its overactivation has been implicated in tissue breakdown and alveolar bone resorption during chronic periodontitis [5,6,12]. The convergence of these mechanisms in ESRD patients, who are already in a state of chronic systemic inflammation, may explain the amplified periodontal response observed in our cohort.

A noteworthy aspect of our results is the apparent divergence between the non-significant omnibus genotype comparison and the significant TT vs. GG contrast. Statistically, such discrepancies are possible when a specific pairwise contrast captures the main genetic effect but the overall χ^2^ test is underpowered to detect differences across all genotype categories [23]. Additionally, multivariable models adjusting for age and sex identified significant genotype effects on both periodontal and renal parameters, indicating that confounding variables can mask associations in unadjusted analyses [24]. FDR correction, although critical to control type I error, inevitably reduces statistical power, a trade-off that is particularly pronounced in exploratory studies with modest sample sizes [24]. The biological plausibility of the TT genotype as a risk factor is supported by previous evidence linking rs2149356 to altered susceptibility in inflammatory diseases, including gout and vascular disorders [15,16,17]. While rs2149356 is intronic, such variants can influence gene function via altered transcription factor binding, splicing efficiency, or chromatin conformation [15,16]. Inflammatory amplification in TT carriers could thus arise from subtle shifts in TLR4 expression or responsiveness to bacterial ligands, leading to heightened cytokine release in the periodontium and systemic circulation. Given that ESRD patients often harbor dysbiotic oral and gut microbiota [6], it is conceivable that a genetic predisposition to stronger TLR4 activation could perpetuate a feed-forward loop of microbial stimulation, systemic inflammation, and tissue destruction.

Clinically, our findings align with the concept of a bidirectional oral–renal inflammatory axis [5,6,7]. Periodontitis can exacerbate systemic inflammation, potentially accelerating CKD progression, while uremia and immune dysregulation in ESRD can worsen periodontal breakdown [6]. These processes are increasingly understood to be mediated by host–microbiota–immune interactions, in which dysbiotic oral biofilms and altered immune responses contribute to systemic inflammatory load and distant organ effects [7,11]. If future studies confirm that TT carriers are more vulnerable to this interplay, genotype-guided risk stratification could inform individualized preventive strategies, such as intensified periodontal surveillance in high-risk ESRD patients. While TLR4-targeted therapeutics remain experimental [13], their potential applicability to both renal and periodontal contexts is noteworthy.

Furthermore, in this population, complementary approaches to traditional non-surgical periodontal therapy (NSPT) might be promising. According to recent research, when paired with NSPT, domiciliary probiotic regimens can enhance periodontal outcomes [32]. Such adjunctive methods could expand the scope of preventive care and connect genotype-informed risk stratification with concrete clinical management strategies when incorporated into periodontal maintenance, especially for genetically susceptible ESRD patients.

Our results on TLR4 rs2149356 reveal both population-specific variations and consistency when viewed in the context of the global genetic landscape. The non-synonymous Asp299Gly (rs4986790) and Thr399Ile (rs4986791) variants, which are uncommon in non-European groups but relatively common in Europeans, have been the subject of the majority of genetic research on TLR4 and periodontitis [15,16]. Although results across European and Asian cohorts have been inconsistent due to ethnic heterogeneity and modest sample sizes, these polymorphisms have been variably linked to altered inflammatory responses and susceptibility to periodontitis. Our Egyptian cohort, on the other hand, demonstrated a detectable frequency of rs2149356 but no presence of these classic variants, which is consistent with previous research in the same population [17].

Notably, rs2149356 has been associated with a higher risk for inflammatory diseases such as gout in Spanish [16] and the Chinese [15] populations, whereas Polynesian cohorts showed a lower risk for the same allele [18]. This implies that the allele’s functional impact could change based on genetic background, environmental exposures, and microbiota. Apart from periodontitis, rs2149356 has been linked to vascular disease [19] and metabolic disorders [20], indicating its broader pro-inflammatory significance.

When combined, these comparisons highlight how TLR4 variants influence host-microbial interactions, but they also highlight how the relevant SNP varies by ancestry. Therefore, our results underline the necessity of population-specific genetic research in under-represented populations to elucidate the role of TLR4 polymorphisms in oral-systemic comorbidities.

Strengths of this study include standardized periodontal assessment by calibrated examiners, blinded genotyping with established 5′ nuclease assays [22], and rigorous statistical handling with both adjusted and unadjusted analyses. However, the study’s limitations must temper interpretation. First, the modest sample size may constrain the statistical power and increase the risk of type II errors, especially for subgroup analysis. Second, the single-centered recruitment limits the generalizability of the results. Third, the cross-sectional design precludes causal inference and temporal ordering between genotype, renal function, and periodontal outcomes. Fourth, even though we made an effort to control for important covariates, residual confounding from elements like medication use, comorbidities, and dialysis duration cannot be eliminated. Fifth, although the study identified a modifying effect for ESRD, the mechanisms, such as the accumulation of uremic toxins and oxidative stress, through which this occurs, are speculative, since we lack biomarker-based evidence for the underlying pathogenesis. Finally, the absence of microbiome profiling and functional assays prevents mechanistic verification of the hypothesized TLR4–microbiota–host interactions. Therefore, future research should address these gaps through larger, multi-center studies with adequate statistical power, robust ancestry adjustment, and longitudinal follow-up to examine genotype-specific trajectories of periodontal and renal disease. Mechanistic work in gingival and renal cell cultures from genotyped donors could clarify whether rs2149356 affects TLR4 expression or signaling thresholds. Multi-omics integration, including transcriptomics, proteomics, and microbiome sequencing, would enable a systems-level understanding of how host genotype interacts with microbial dysbiosis in ESRD [6,7,12]. Such studies could ultimately inform targeted interventions at the intersection of dentistry and nephrology.

## 5. Conclusions

In summary, our exploratory data suggest that the TLR4 rs2149356 TT genotype may be associated with an increased inflammatory burden in ESRD patients, manifesting in both periodontal and renal parameters. While genotype distributions were not significantly different after FDR correction, converging signals from relative risk estimates and adjusted regression models merit further investigation. Confirmation in larger, mechanistically informed studies will be essential before considering clinical application of genotype-based periodontal risk management in ESRD.

## Figures and Tables

**Table 1:** Sociodemographic characteristics of ESRD and control groups.

Characteristic	ESRD (n = 46)	Control (n = 46)	Statistical Test	*p*-Value
Age (years)	49.91 ± 11.62 Median: 48 (38–60)	52.93 ± 11.97 Median: 55 (30–60)	*t*-test	0.225
Gender			χ^2^	0.135
Male	28 (60.8%)	34 (73.9%)		
Female	18 (39.2%)	12 (26.1%)		
Educational level			χ^2^	0.690
High education	12 (26%)	14 (30%)		
Primary education	28 (61%)	24 (52%)		
Illiterate	6 (13%)	8 (17%)		
Financial income			χ^2^	0.865
Fixed income	31 (67%)	29 (63%)		
No fixed income	12 (26%)	14 (30%)		
No income	3 (7%)	3 (7%)		

Values are presented as mean ± standard deviation (SD) and median (range) for continuous variables, and as frequency (percentage) for categorical variables. *t*-test = Student’s *t*-test; χ^2^ = Chi-square test; ns = non-significant.

**Table 2:** Comparison of genotype frequency of TLR4 rs2149356 polymorphism in ESRD and control groups (χ^2^ test, FDR-adjusted).

Group	GG n (%)	GT n (%)	TT n (%)	Total n (%)	χ^2^	Raw *p*-Value	FDR-Adjusted *p*-Value/OR (GG vs. TT)
ESRD	12 (26.67%)	13 (28.89%)	20 (44.44%)	45 (49%)	5.0826	0.079	0.118/≈3.90
Control	4 (8.70%)	16 (34.78%)	26 (56.52%)	46 (51%)			
Total	16 (17.58%)	29 (31.87%)	46 (50.55%)	91 (100%)			

χ^2^ = Chi-square test; OR = Odds ratio; FDR = False discovery rate. Adjusted *p* ≈ 0.118 > 0.05, ns = non-significant.

**Table 3:** Comparison of allele frequency of TLR4 rs2149356 polymorphism in ESRD and control groups (χ^2^ test, FDR-adjusted).

Group	G Allele n (%)	T Allele n (%)	χ^2^	Raw *p*-Value	FDR-Adjusted *p*-Value	OR (G vs. T)
ESRD	37 (41%)	53 (59%)	3.959	0.0466	0.093	≈1.98
Control	24 (26%)	68 (74%)				
Total ^1^	61 (33%)	121 (67%)				

^1^ Percentages in the Total row are calculated from the combined allele counts across both groups (n = 182 alleles). The ESRD group total is based on 90 alleles due to incomplete genotyping for 2 alleles. χ^2^ = Chi-square test; OR = Odds ratio; FDR = False discovery rate. Adjusted *p* ≈ 0.093 > 0.05, ns = non-significant.

**Table 4:** Relative risk (RR) of ESRD for different TLR4 rs2149356 genotypes.

Comparison	Genotype	ESRD n (%)	Control n (%)	RR	95% CI	Raw *p*-Value	FDR-Adjusted *p*-Value
GT vs. GG	GT	13 (28.89%)	16 (34.78%)	1.91	1.06 to 3.43	0.030 *	0.060
	GG	12 (26.67%)	4 (8.7%)				
TT vs. GG	TT	20 (44.44%)	26 (56.52%)	3.25	1.90 to 5.59	0.010 *	0.020 *
	GG	12 (26.67%)	4 (8.7%)				

RR = Relative risk; CI = Confidence interval; FDR = False discovery rate; * *p* ≤ 0.05.

**Table 5:** Comparison of periodontal parameters by genotype in control vs. ESRD groups.

Parameter	Genotype	Control Mean ± SD	ESRD Mean ± SD	FDR-Adjusted *p*-Value
Gingival Index (GI)	GG	1.75 ± 0.50	2.83 ± 0.39	0.030 *
	GT	1.93 ± 0.25	2.69 ± 0.48	0.0002 *
	TT	1.92 ± 0.27	2.65 ± 0.50	0.0002 *
Plaque Index (PI)	GG	0.29 ± 0.09	0.29 ± 0.05	0.950
	GT	0.29 ± 0.08	0.26 ± 0.09	0.523
	TT	0.27 ± 0.08	0.27 ± 0.07	0.865
Plaque %	GG	0.50 ± 0.18	0.45 ± 0.13	0.793
	GT	0.43 ± 0.09	0.44 ± 0.11	0.857
	TT	0.52 ± 0.17	0.46 ± 0.14	0.293
BOP %	GG	50 ± 18%	46 ± 12%	0.857
	GT	43 ± 10%	44 ± 11%	0.857
	TT	52 ± 17%	46 ± 14%	0.293
Deepest PD (mm)	GG	5.00 ± 1.00	5.00 ± 0.74	1.000
	GT	5.24 ± 1.26	5.46 ± 1.76	0.838
	TT	5.27 ± 1.75	6.40 ± 1.98	0.066
Deepest CAL (mm)	GG	5.00 ± 1.00	5.25 ± 0.78	0.887
	GT	5.24 ± 1.26	5.46 ± 1.76	0.838
	TT	5.27 ± 1.75	6.40 ± 1.98	0.066

FDR = False discovery rate; GI = Gingival index; PI = Plaque index; BOP = Bleeding on probing; PD = Probing depth; CAL = Clinical attachment loss. * *p* ≤ 0.05.

**Table 6:** Multivariable regression for TT vs. GG genotype, adjusted for age and gender, combining renal and periodontal parameters (n = 46).

Outcome Variable	β (TT vs. GG)	SE	*p*-Value
Probing Depth (PD)	1.4	0.58	0.033 *
Clinical Attachment Loss (CAL)	1.13	0.55	0.033 *
Gingival Index	0.9	0.21	<0.001 *
Plaque %	–0.06	0.04	0.176 ns
Bleeding on Probing (BOP %)	–6.00	4.35	0.176 ns
ln(Serum Creatinine)	0.31	0.12	0.012 *
ln(Blood Urea)	0.27	0.1	0.009 *

SE = Standard error; β = Regression coefficient; PD = Probing depth; CAL = Clinical attachment loss; BOP = Bleeding on probing; * *p* ≤ 0.05; ns = non-significant.

**Table 7:** Inter-examiner reliability for periodontal clinical parameters.

Parameter	Reliability Coefficient (95% CI)	*p*-Value
Mean PD	0.982 (0.971–0.990) ^1^	<0.001 *
Plaque %	0.955 (0.932–0.971) ^1^	<0.001 *
BOP %	0.962 (0.943–0.975) ^1^	<0.001 *
Deepest CAL	0.988 (0.979–0.994) ^1^	<0.001 *
Gingival index (mean)	0.886 (0.842–0.918) ^2^	<0.001 *

^1^ ICC(2,1), two-way random effects, absolute agreement. ^2^ Weighted Cohen’s kappa (quadratic weights) for ordinal scores (gingival index). * Significant at *p* < 0.05.

## Data Availability

The original contributions presented in this study are included in the article. Further inquiries can be directed to the corresponding author.
